# 

**Published:** 1916-10

**Authors:** 


70th ANNIVERSARY YEAR

LIBRARY
OCT. 30. 1916
SURGEON-GENERAL'S OFFICE

### THE DENTAL REGISTER

Edited by Dr NELVILLE S. HOFF
ANN ARBOR, MICH.

VOL. LXX OCTOBER, 1916 NO. 10

THE SAMUEL A. CROCKER CO., Publishers
OHIO DENTAL AND SURGICAL DEPOT
CONRAD BLDG., 18 & 20 W. Seventh St., CINCINNATI, O.

PRICE, ONE DOLLAR A YEAR

Entered at the Post-Office at Cincinnati, O., as second-class mail matter

